# Fibers Generated by Plasma Des-AA Fibrin Monomers and Protofibril/Fibrinogen Clusters Bind Platelets: Clinical and Nonclinical Implications

**DOI:** 10.1055/s-0041-1725976

**Published:** 2021-07-06

**Authors:** Dennis K. Galanakis, Anna Protopopova, Liudi Zhang, Kao Li, Clement Marmorat, Tomas Scheiner, Jaseung Koo, Anne G. Savitt, Miriam Rafailovich, John Weisel

**Affiliations:** 1 Department of Pathology, Stony Brook University School of Medicine, Stony Brook, New York; 2 Department of Cell and Developmental Biology, University of Pennsylvania School of Medicine, Philadelphia, Pennsylvania; 3 Department of Materials Science and Engineering, Stony Brook University, Stony Brook, New York; 4 Department of Microbiology and Immunology, Stony Brook University School of Medicine, Stony Brook, New York

**Keywords:** fibrin fibers, fibrinogen adsorption, soluble fibrin, cryoprecipitate fibrinogen, protofibril/fibrinogen clusters, platelet adhesion

## Abstract

**Objective** Soluble fibrin (SF) is a substantial component of plasma fibrinogen (fg), but its composition, functions, and clinical relevance remain unclear. The study aimed to evaluate the molecular composition and procoagulant function(s) of SF.

**Materials and Methods** Cryoprecipitable, SF-rich (FR) and cryosoluble, SF-depleted (FD) fg isolates were prepared and adsorbed on one hydrophilic and two hydrophobic surfaces and scanned by atomic force microscopy (AFM). Standard procedures were used for fibrin polymerization, crosslinking by factor XIII, electrophoresis, and platelet adhesion.

**Results** Relative to FD fg, thrombin-induced polymerization of FR fg was accelerated and that induced by reptilase was markedly delayed, attributable to its decreased (fibrinopeptide A) FpA. FR fg adsorption to each surface yielded polymeric clusters and co-cryoprecipitable solitary monomers. Cluster components were crosslinked by factor XIII and comprised ≤21% of FR fg. In contrast to FD fg, FR fg adsorption on hydrophobic surfaces resulted in fiber generation enabled by both clusters and solitary monomers. This began with numerous short protofibrils, which following prolonged adsorption increased in number and length and culminated in surface-linked three-dimensional fiber networks that bound platelets.

**Conclusion** The abundance of adsorbed protofibrils resulted from (1) protofibril/fg clusters whose fg was dissociated during adsorption, and (2) adsorbed des-AA monomers that attracted solution counterparts initiating protofibril assembly and elongation by their continued incorporation. The substantial presence of both components in transfused plasma and cryoprecipitate augments hemostasis by accelerating thrombin-induced fibrin polymerization and by tightly anchoring the resulting clot to the underlying wound or to other abnormal vascular surfaces.

## Introduction

Blood bank plasma contains substantial levels of soluble fibrin (SF) that generate its cryoprecipitate, and this contains up to one-third of plasma fibrinogen (fg) in the form of SF 1 2 which is also a substantial component of isolated fg. 2 Apart from increased SF levels in 8 to 13% of hospitalized patients, 3 reports intimated possible SF roles in the fg link to inflammation 4 and in thrombotic complications of intravascular devices, 5 fg replacement therapy, 6 and multiple perioperative transfusions. 7 In the present study, we investigated the possible SF role in the incompletely understood fiber generation by fg. 8 9

Fg is a 342 kDa major plasma glycoprotein which consists of three pairs of disulfide-linked nonidentical polypeptide chains, Aα, Bβ, and γ. It is assembled in two identical halves linked at their central (or E) region that contains the amino termini of all six chains. 10 Each outer or D region contains the carboxyl terminal of each chain, and that of Aα chains is the longest and is tethered to the E region. This part is known as the αC region encompassing residues Aα221–610, which include the αC connector (Aα221–391) and the αC domain (Aα392–610). 11 Thrombin cleaves two pairs of small N-terminal fibrinopeptides, FpA, and FpB, from its Aα and Bβ chains, respectively (on the E region). The cleavages expose pairs of “A” and “B” binding sites, known as “knobs” (α17–19 and β15–18, respectively), which interact with “holes” or “pockets” termed as “a” (γ337–379) and “b” (β397–432) sites and located in each D region. The ensuing complimentary (knob/hole) binding initiates the two molecule-thick fibril (protofibril), which elongates by staggered monomer assembly. During this phase, protofibrils are soluble with little or no detectable turbidity. 12 Elongated fibrils undergo lateral contacts initiating generation of insoluble fiber networks with detectable turbidity and varying fiber thickness and branching. 10 The network is stabilized by thrombin-activated plasma transglutaminase (F XIII) that catalyzes formation of covalent γ-glutamyl-ε-lysyl intermolecular crosslinks. 13 Four such links form on each α and two on each γ chain.

We examined atomic force microscopy (AFM) images of cryoprecipitable SF-rich fg subfraction adsorbed to a hydrophilic 14 and to two hydrophobic 8 surfaces, the latter more extensively used. Effects of hydrophobic fg adsorption reportedly include tight binding mostly via its D regions whose diameters are increased and heights are decreased, 15 16 binding is tighter after thrombin treatment, 17 fibrinopeptide A (FpA) cleavage, 18 and coagulability 19 are unimpaired, and cryptic fg epitopes that bind PAI-I, 20 MAC, 21 and Aα 95–97 (RGD) 22 are exposed. Our results revealed that SF accounted for fiber generation by fg and that resulting fibers bound platelets. What is more, we identified a major SF component comprising des-AA monomers whose adsorption initiated protofibril generation and promoted elongation. Additionally, a minor SF component consisted of multimeric clusters with a protofibril core whose adsorption also promoted protofibril generation.

## Materials and Methods

### Reagents, Supplies, and Related Procedures

Except where otherwise specified, reagents were purchased from Fisher Scientific (Springfield, New Jersey, United States). Alexa Fluor 594 conjugated goat antimouse immunoglobulin (Ig)-G1 and hexamethyldisilazane were purchased from Thermo Fisher Scientific (Waltham, Massachusetts, United States). Purchased from Sigma–Aldrich (St. Louis, Missouri, United States) were human and bovine serum albumin, glutaraldehyde, ethylenediaminetetraacetate (EDTA), phenylmethylsulfonylfluoride (PMSF), GlyProArgPro (GPRP) peptide, HEPES and imidazole buffers, and salts for other buffers and for fg precipitation. Buffers routinely containing 0.14 M NaCl included 0.01 M Na _2_ PO _4_ , pH 6.4 (phosphate buffered saline) and Tris-HCl, pH 7.4 (Tris-HCl buffered saline [TBS]) with or without 0.05% NaN _3_ . D-phenyl-L-prolyl- L -arginine chloromethylketone (PPACK) and dithiothreitol were purchased from Calbiochem-Behring (La Jolla, California, United States). Factor XIII (F XIII) was purchased from Enzyme Research (South Bend, Indiana, United States). Obtained as described 23 were human thrombin and afibrinogenemic plasma. Reptilase was purchased from Pentapharm (Basel, Switzerland). Monoclonal antibodies (mAbs) against Aα241–476, Aα518–584, and γ86–411 were kind gifts by Dr. Bohdan Kudryk. As described previously, 8 24 3 mm thick silica wafers were coated with either trioctylmethylamine (TOMA) or polystyrene (PS) yielding a coating height approximately 100 nm, and respective water droplet angles of 69 ± 7 degrees, *n* = 6, and 91 ± 1 degrees. Each wafer was cut into 1 cm ^2^ plates and exposed to solutions of fg isolates (vide infra). For viscoelastometry, 2 mg fg/mL (pH = 7.4) was clotted with thrombin 0.5 U/mL and assessed by using the Malvern Rotational Rheometer. Precast electrophoresis (SDS-PAGE) gels were from Bio-Rad (Hercules, California, United States). Performed as described were isolation of gel-sieved platelets 25 and fibrinopeptide measurements. 26 For fiber viscoelastometry, the AFM tip was used to indent the fiber with a constant force applied to maintain contact. The G' was computed by use of the mechanical responses and a set of related parameters. 8

### Fg and Fibrin Procedures

Fg, >98% coagulable, was isolated by the glycine procedure 27 from human plasma pools of at least 10 donors. Selected pools had been cryoprecipitate-depleted. FG was reprecipitated with 25% saturated (NH _2_ ) _2_ SO _4_ to remove (floating) lipid aggregates. The fg precipitate was dissolved and dialyzed in 0.3 M NaCl, treated with 10 nM of either PMSF or PPACK for 30 minutes or stored untreated in small aliquots at −70 degrees. Chromogenic assay 28 of four untreated isolates yielded no thrombin activity. Performed as described 27 29 30 31 were spectrophotometric fg measurements, extinction coefficient of 15.5 (280 nm, 1 cm, 1%), electrophoresis in dodecyl sulfate polyacrylamide (SDS-PAGE), preparation fg fragment D _1_ , and isolation of des-αC fg. Estimated from the size of its Aα core remnants, des-αC fg (old term fraction I-9) 29 30 and from determinations of C-terminal residues (Asn-269, Gly-297, and Pro-309), 32 such isolates lacked virtually all their αC-domain and variable C-terminal parts of their αC-connector. To prepare FR (fibrin-rich) fg, isolates were dialyzed thrice at 4 degrees versus 0.05 M Na _2_ PO _4_ buffer, pH of 6.4 for intact fg, and 0.02 M for des-αC fg. Precipitates (FR fg) were harvested by centrifugation (4 degrees), and supernatants (FD fg) were concentrated by 25% saturated (NH _2_ ) _2_ SO _4_ reprecipitation and dialysis. Both were stored frozen (vide supra) and thawed rapidly, 37 degrees for use. FR fg was also prepared by exposing plasma cryoprecipitate in situ to ice cold PBS buffer excess overnight three times, dissolving, and storing in 0.3 M NaCl. By SDS-PAGE, some FR fg isolates displayed trace amounts of γ-γ dimers which were absent in fg isolated from cryoprecipitate-depleted plasma. All FR fg isolates were crosslinked, when needed, by 2 mM CaCl _2_ and 10 μM F XIII for 60 minutes or overnight (but displayed some noncrosslinked monomers consistent with another report 33 ). To concentrate clusters, FR fg isolates were subjected to either centrifugation at 7,000 × G, 20 minutes, or treated with 30 mM CaCl _2_ for 60 minutes or overnight. Resulting minor precipitates, termed FRC were dissolved and dialyzed in 0.3 M NaCl, amounted to 7 to 21% ( *n* = 4) of the total protein and stored frozen (vide supra). Fibrin polymerization, monitored by turbidity, 340 nm, was induced by thrombin 0.2 U/mL or reptilase 1 U/mL, pH of 6.4, fg 1 mg/mL. For screening, some mixtures were monitored visually up to 2 hours for possible clot formation.

### Optical and Atomic Force Microscopy

A metallurgical (Olympus) microscope was used for optical microscopy of fg adsorbed to PS-coated silica wafers. 9 To quantify images, a superimposed grid, 34 consisting of 50 × 50 μm ^2^ , was applied and fiber containing squares were expressed as % of the 1 cm ^2^ area. For conventional AFM, an ICON scanning probe microscopy (SPM, Bruker, Santa Barbara, California, United States) was used in both topographic and friction modes. 14 To generate a ≤100 nm thick, mostly monomeric fg layer on PS surface either 50 to 100 µg/mL fg, TBS, 0.5 mM EDTA, for 1 to 2 hours, or 2 to 4 mg/mL fg for 1 minute were used. Longer incubation (>1 hour) of the latter generated fibers as described. 24 For inhibitor effects, the monomeric layer was exposed for 60 minutes to TBS containing 5 µM fragment D _1_ , or 10 μM GPRP peptide, or 1% defatted human albumin. After incubation and supernatant removal, 3 mg/mL FR fg was applied overnight, followed by washing, air-drying, 24 and examination by optical microscopy (vide supra).

For enhanced AFM resolution, a modified graphite (MG) surface (water droplet angle 45.5 ± 0.5 degrees, *n* = 3) was used. 35 A 2 μL aliquot of fg 5 μg/mL solution was applied for 5 to 15 seconds immediately diluted with 100 μL deionized water for 10 seconds dried by forced air, and scanned by AFM in tapping mode using super-sharpened cantilevers, SSS-SEIHR (Nanosensors, Germany) with a tip radius <0.5 nm, and the MFP-3D AFM microscope (Asylum Research, Oxford Instruments, United States). For protofibril preparation, to FR fg 20 μg/mL in HEPES buffer pH 7.4 containing 5 mM CaCl _2_ , thrombin was added to 0.05 U/mL. At 6 minutes, a 2 μL aliquot was subjected to the foregoing MG adsorption and AFM scanning.

### Scanning Electron Microscopy

To evaluate thrombin-induced clots and platelet binding to adsorption induced fibers, scanning electron microscopy was employed. 9 36 Gel-sieved platelets 105 × 10 ^3^ /μL TBS-EDTA buffer (vides supra) were applied to fibers preformed on PS coated wafers and placed on a shaker at 60 rpm for 30 minutes. After washing with buffer wafers were subjected to fixation by 2% glutaraldehyde (v/v in TBS) for 12 hours at ambient temperature. They were subsequently dehydrated by standard stepwise increase of ethanol concentrations and hexamethyldisilazane, air-dried overnight, gold sputter-coated (45 seconds, and examined by a scanning electron microscope (JEOL USA, Inc., Peabody, Massachusetts, United States) at accelerating voltage from 5 to 15kV.

### Confocal Microscopy

To evaluate exposed fg epitopes anti-Aα241–476, anti-Aα518–584 and anti-γ86–411 mAbs were each applied at 100 μM for several hours. Supernatants were removed and plates were washed three times with buffer excess. To demonstrate mAb binding, the Alexa Fluor 594 conjugated goat anti-mouse lgG1 antibody was applied for 1.5 hours at ambient temperature. The microplates were then washed and examined by the TCS SP8 X laser scanning confocal microscope (Leica Microsystem, Buffalo Grove, Illinois, United States). Bovine albumin applied as 0.5% solution to PS microplates was used as negative control.

### Declaration of Helsinki

This study used pooled blood bank plasma from unidentified blood donors whose standard permission for blood donation included its possible use for research, and this complied with the Helsinki Declaration of Ethical Principles.

## Results

### General Characteristics of FR fg

**Fig. 1:**
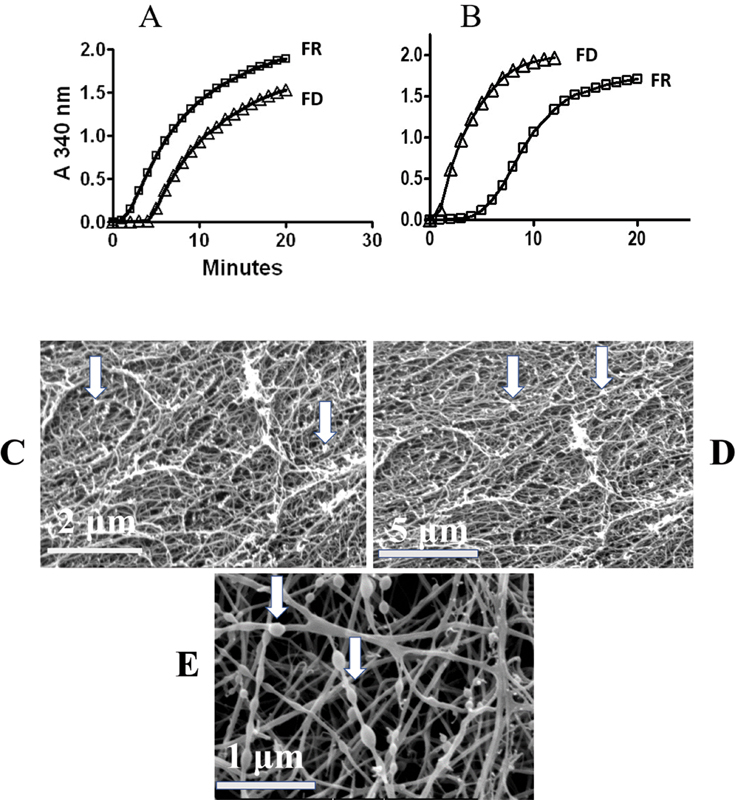
**Polymerization time course and clot imaging.** ( **A** ) Thrombin-induced fibrin polymerization showing a faster onset by FR than by FD fg. ( **B** ) Reptilase induced polymerization showing a marked onset delay by FR fg. ( **C** ) Scanning electron micrograph of a thrombin induced clot of isolated fg (2 mg/mL, not subfractionated into its FD and FR components), showing small globules peppered throughout the field and attached to fibers, arrows, magnification bars shown = 2, 5, and 1 μm for C, D, and E, respectively. ( **D** ) Scanning electron micrograph of a clot from a citrated single donor normal plasma, fg 2.5 mg/mL, also showing such nodules, arrows, magnification bar = 5 μm. ( **E** ) Scanning electron micrograph of a clot from fg, 2 mg/mL, of a thrombophilia patient showing two distinct nodule groups, magnification bar = 1 μm; in one group clusters appear embedded within the fibers along their length like beads on a string, right arrow. In the other group, they appear attached to the fiber surface, left arrow. FD, fibrinogen depleted of soluble fibrin; Fg, fibrinogen; FR, fibrinogen enriched with soluble fibrin.

The FR fg content of different fg isolates, including two obtained commercially and several from cryoprecipitate-depleted plasma, was measured by spectrophotometer and yielded 36 ± 4%, *n* = 9, of the parent isolates. Two des-αC isolates yielded FR of 5 and 8%, which also contained the minor amounts of fg with intact Aα chains. 29 To be sure, these FR fg measurements reflected both SF/fg complexes 37 and cryoprecipitable fibrin monomers. 2 To ascertain if cryoprecipitability reflected fibrin polymerization, two separate experiments were performed by using 10 mg/mL FR fg. One mixture contained fragment D _1_ in three molar excess, and another 1% defatted human albumin (that potentiates fg inhibition of fibrin polymerization 34 ). After 30 minutes, the vortexed mixtures were subjected to the cryoprecipitation procedure (see materials and methods). No precipitate formed in either mixture, in sharp contrast to untreated FR fg controls, indicating that cryoprecipitability did reflect fibrin polymerization. Four lines of evidence indicated that des-AA fibrin was a major fibrin species in SF. One was thrombin-released fibrinopeptides 26 from FR fg that yielded 64% FpA and undiminished FpB, in general agreement with other reports. 2 38 Another was the accelerated thrombin-induced polymerization, Fig. 1A consistent with another report 37 and attributable to pre-exposed knobs A. A third line of evidence was the delayed reptilase induced polymerization, Fig. 1B implying decreased available FpA bonds. Still another line of evidence was the substantial fg populations whose cryoprecipitability reflected des-AA monomers. In viscoelastic comparisons, G' of an FR fibrin clot induced by thrombin, was 81.5 (81–83, *n* = 3) MPa and that of an FD clot was 111.5 (111–113) MPa. Corresponding stress break points were 48 MPa (FR) and 78 MPa (FD). The decreased G' possibly reflected structural differences between the two networks. For example, areas of fine fibril networks with minimal if any branching (vide infra) may display decreased rather than increased 39 G'.

**Fig. 2:**
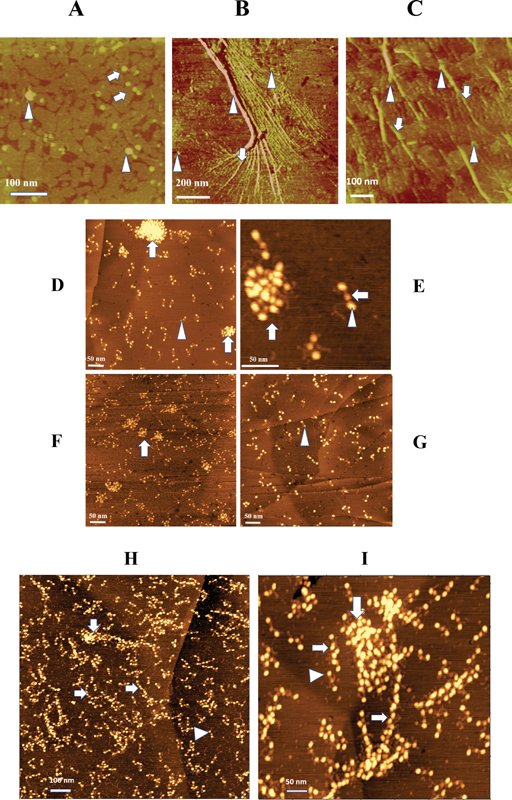
**Atomic force microscopy images of fg clusters, fibrils, and fibers.** ( **A** ) Topographic TOMA field of des-αC fg, overnight adsorption time, showing trinodular monomers, arrows, no fibers, and numerous solitary globules of which one or more suggests an undulating surface, left arrowhead, and another suggests possible merging, right arrowhead. ( **B** ) A lateral TOMA scan (defined by decreased surface resistance to the scanning probe and) obtained following overnight adsorption of FR fg, modified from. 8 It shows numerous fibrils in parallel orientation with embedded and closely spaced nodules along the fibril length, arrow. Some fibrils (likely protofibrils) appear linked to the underlying surface and are merging to form coarser fibrils of varying width. These appear to merge further to form the coarse fiber, middle arrowhead, also displaying numerous solitary nodules on its surface. Left and right arrowheads point to two of the many other, fiber-linked solitary nodules. The three dark oblong spots are technical artifacts. ( **C** ) Topographic TOMA field of FR fg from 5 minutes. adsorption time showing fibers of limited length with numerous embedded nodules along their lengths, arrows, and others that appear attached to fibers, arrowheads. ( **D** ) An MG field of FR fg, adsorption time <15 seconds showing numerous adsorbed monomers, arrowhead, and two clusters, arrows. ( **E** ) Higher magnification of an MG field of adsorbed FR fg showing one cluster, left arrow, and regions D (arrowhead) and E (right arrow) of a monomer. ( **F** ) MG field showing adsorbed fg from cryoprecipitate (see materials and methods) with numerous monomers and many variable size clusters, arrow. ( **G** ) An MG field showing a typical area of adsorbed FD fg with monomers and no clusters. ( **H** ) An MG field of thrombin-treated FR fg showing numerous adsorbed protofibrils, horizontal arrows, monomers, arrowhead, and a small cluster, vertical arrow that appears enveloped by at least one protofibril. ( **I** ) A similar MG field of thrombin-treated FR fg showing two clusters, one indicated by the vertical arrow, linked to short and long protofibrils, indicated by the left and the right horizontal arrow. A monomer is shown by the arrowhead. Note that the size and density of each D region, arrowhead, and those of protofibrils, horizontal arrows, appear indistinguishable from one another.

A different series of experiments focused on nodules observed in images of clots and among images of adsorbed fg. Thrombin-induced clots Fig. 1C and D displayed nodules peppered across surfaces and were invariably linked to fibers, and in a patient clot as shown in Fig. 1E , some appeared embedded in fibers. In adsorbed fg, Fig. 2A nodules typically appeared among monomers in some fields, and with fibers in others such as shown in Fig. 2B and C (arrowheads). Moreover, the larger nodules displayed regularly undulating margins ( Fig. 2A , arrowheads), reflecting their polymeric composition (vide infra). Lastly, the nodules of des-αC fg, Fig. 2A indicated that intact αC was not required for their formation.

### Demonstration of Multimeric Clusters and Their Relationship to Protofibrils

For a more detailed examination of the nodules, an increased resolution AFM procedure 14 using the MG surface was employed. To minimize possible adsorption effects, the fg solution was exposed to the MG surface for ≤15 seconds. AFM scans ( Fig. 2D–F , vertical arrows) disclosed multimeric clusters along with an abundance of solitary monomers in FR fg, as illustrated in Fig. 2D (arrowhead). Moreover, fg regions D and E, as shown in Fig. 2D (arrowhead) and Fig. 2E (horizontal arrow), were clearly distinguishable by their size and density. Notwithstanding somewhat variable cluster shape, measurements of the widest diameter among a selection of smaller clusters yielded a range of 71 to 155 nm ( *n* = 5). Additionally, the abundance of solitary monomers that coisolated with clusters ( Fig. 2D and F ), underscored their cryoprecipitability. In sharp contrast, FD fg scans showed fields of monomers ( Fig. 2G ) with rare clusters (not shown). Moreover, when the adsorption time was prolonged (i.e., >10 minutes), no additional images emerged. Also, estimates in five of the smallest clusters yielded a range of 9 to 11 D and E regions, representing approximately five monomers/cluster.

In an additional experiment, advantage was taken of the adsorption of thrombin-induced protofibrils to the MG surface 14 to explore the relationship of clusters to protofibrils. As shown, protofibrils were closely linked to small clusters ( Fig. 2H and I , vertical arrows). Each cluster, moreover, appeared partly unraveled with some of its monomers being parts of the adjoining protofibrils, notwithstanding that the alternative possibility of cluster formation could not be excluded. Additionally, the D region of monomers ( Fig. 2H and I , arrowheads) appeared indistinguishable from the DED complex along the protofibril length (horizontal arrows panel I). This similarity served to identify protofibrils in subsequent experiments.

### Crosslinking Analyses of Clusters

**Fig. 3:**
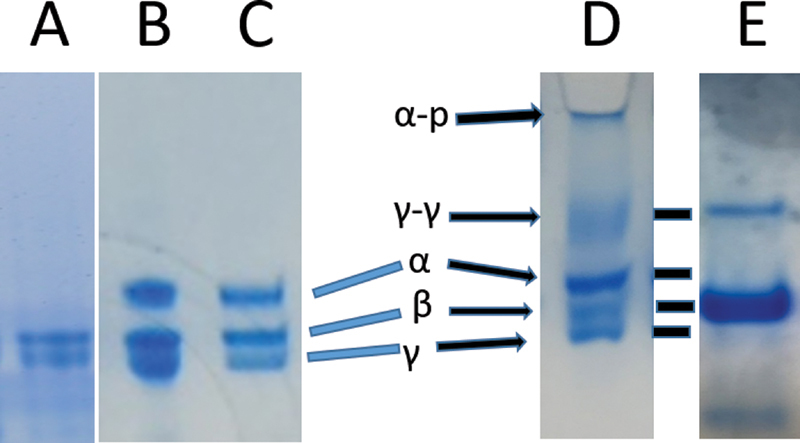
**SDS-PAGE gels of dithiothreitol-reduced fg.A–C** show untreated des-αC, FR, and FD fg, respectively. **Lane D:** FR fg showing γ-γ and α-polymer bands along with noncrosslinked γ and α chain bands. The sample had been incubated overnight with 30 mM CaCl _2_ . **E lane** : an aliquot from a cluster concentrate (see materials and methods) showing more pronounced γ-γ and α-polymer bands and virtual absence of noncrosslinked α and γ chain bands. Each lane is from a separate gel run except for B and C. The corresponding molecular weights (expressed in Daltons × 10 ^−3^ and rounded off to the nearest whole number) were α-polymer, 400 (this polymer band does not penetrate the 4 to 15% polyacrylamide gel top used unless incompletely crosslinked), γ-γ dimer 105, α chain 71, β chain 60, and γ chain 51.

We reasoned that crosslinking conditions may be informative since they typically do not crosslink solitary fg monomers, save at suggested high concentrations (e.g., fg 30 mg/mL). 33 Accordingly, we took advantage of trace amounts F XIII usually present in fg isolates 13 by subjecting FR fg to 30 mM CaCl _2_ ( *n* = 5). Indeed, examination by SDS-PAGE consistently disclosed formation of major γ-γ dimer and α-polymer (gel origin) bands copresent with those of noncrosslinked counterpart chains ( Fig. 3D ), in contrast to untreated controls ( Fig. 3A–C ). At least several cluster concentrates, moreover, disclosed crosslinking of all cluster components ( Fig. 3E ) indicating that fg/fibril cluster contacts included αC/αC apart from single knob/hole counterparts. Also, the virtual absence of γ-γ dimers in some crosslinked isolates, not shown, possibly reflected trace amounts of tissue transglutaminase that typically catalyzes α–γ crosslinks.

### Time Course of Fiber Generation

**Fig. 4:**
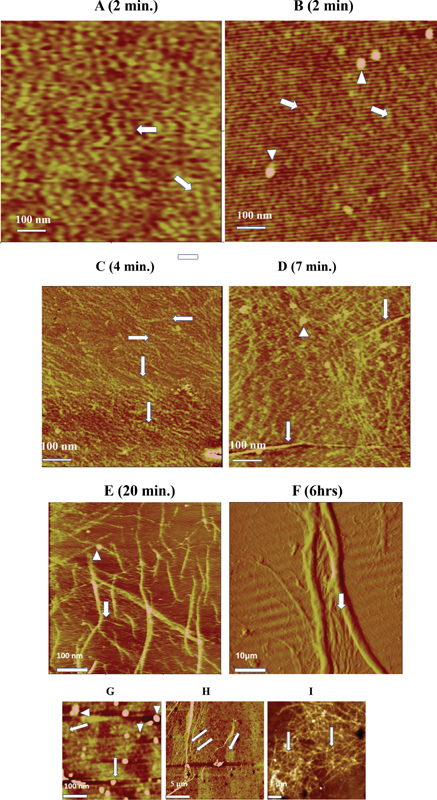
**Atomic force microscopy images resulting from timed adsorption to PS or TOMA surface of FR fg and its subfraction FRC.** ( **A** ) Lateral scan from a 2-minute TOMA adsorption time showing a layer with many trinodular images (monomers), horizontal arrow, and some multinodular (>3 nodule long) linear images, oblique arrow, each indicating a protofibril. Other images show one or two fg (D or E) regions reflecting the random orientation of monomers in the adsorbed layer. ( **B** ) Topographic scan from another 2 minutes TOMA adsorption time also showing numerous protofibrils, oblique arrows, along with clusters appearing as dense nodules with undulating margins, arrowheads. ( **C** ) Topographic scan from 4 minutes PS adsorption time, showing more numerous and longer protofibrils, horizontal arrows, in somewhat parallel orientation and at least two double protofibril images, vertical arrows. Also shown are occasional clusters appearing linked to protofibrils, one under the top arrow. ( **D** ) Topographic scan from 7 minutes PS adsorption time, showing increased protofibrils with some in parallel and others in random orientation. Also shown are two fibers, vertical arrows, and clusters of varying size and undulating surfaces, arrowhead, appearing closely linked to protofibrils and fibers. ( **E** ) Topographic scan following 20 minute PS adsorption time and showing overlapping fibers with some branching, and protofibrils linked to fibers, arrow. Clusters are again shown with varying size and closely linked to protofibrils and fibers, arrowhead. ( **F** ) Lateral scan from a 6-hour TOMA adsorption time showing many variably fine and two coarse fibers, modified from our previous report. 8 Note that fine fibers appear to rise from the surface and wrap around their course counterparts, arrow. The horizontal dark and light background lines are mechanical artifacts. ( **G** ) Topographic scans of 2 minutes PS adsorption time of cluster concentrate, FRC, showing numerous variable size clusters, vertical arrowheads, some overlapping or confluent, horizontal arrowhead, and protofibrils, arrows. ( **H** ) Topographic scan of the same concentrate following 15-hour PS adsorption time, showing fine fibrils converging toward and many linked to a fiber, and clusters linked to fibrils, left arrows. Other fine fibrils appear in a solitary patch, right arrow. ( **I** ) Topographic scan of 15-hour PS adsorption time showing a solitary network patch consisting of uniformly fine fibrils almost all with embedded clusters appearing like beads on a string, arrows. Repeated for clarity, magnification bars for G, H, and I are 100 nm, 5 μm, and 1 μm, respectively.

First, evidence of fiber generation induced by adsorption to various hydrophobic surfaces 8 9 24 raised the question of mechanism, which led to the present time course experiments monitored by AFM. At 2-minute adsorption time trinodular monomers, appeared Fig. 4A , (horizontal arrow), along with occasional linear images of more than three nodules, oblique arrow, each indicating a protofibril. Additionally, many monomers showed less than three nodules reflecting random orientation within the (100-nm thick) layer. 8 Second, a 2-minute experiment focused on a field area displaying a few large solitary nodules whose undulating surface margins indicated multimeric clusters ( Fig. 4B , arrowheads) in addition to numerous protofibrils (arrows). At 4-minute adsorption time, protofibrils appeared longer and more numerous and randomly distributed ( Fig. 4C , horizontal arrows) and at least two appeared in closely parallel positions ( Fig. 4C , vertical arrows). At 7-minute adsorption time, areas of parallel protofibril orientation emerged along with long fibers ( Fig. 4D , vertical arrows and occasional clusters, arrowhead). At 20 minutes, fibers were more numerous with variable diameter widths and overlaps, indicating above-surface networks and branching ( Fig. 4E , arrow and occasional clusters, arrowhead). At 6 hours, numerous fibrils of varying width were linked to coarse fibers ( Fig. 4F , vertical arrow) and many fibrils appeared wrapped around fibers resulting in irregular fiber margins as shown.

### Examination of a Cluster Concentrate’s Fiber Generation Capacity

Our aim here was to assess by AFM if clusters per se obtained from FR fg lacking F XIII (see materials and methods) could generate fibers. First, a single functional residual capacity (FRC) isolate was incoagulable by reptilase, *n* = 3, indicating absent or inaccessible fg. Second, three isolates displayed numerous clusters at 2 minutes adsorption time to PS ( Fig. 4G , arrowheads, and short protofibrils, arrows). Third, following several or more hours of adsorption time numerous fibrils linked to fibers ( Fig. 4H , left arrows appeared). Fourth, a minor but distinct fibril network patch was also evident ( Fig. 4H , right arrow). Such networks appeared as unorganized tangles ( Fig. 4I ) and displayed virtually identical fibril widths (diameters between nodule spans) which were similar to those of protofibrils ( Fig. 4G , arrows). They clearly differed from those of the parent FR fg ( Fig. 2B ), which were also nonbranching but long and in parallel orientation and displayed protofibrils merging to form fine fibrils which in turn appeared merged to form coarse fibers.

### Fiber Properties

We reported that fg fibers generated by hydrophobic adsorption displayed banding periodicity and G' which were similar to counterparts of thrombin-induced fibers. 8 Our present viscoelastic measurements of single fibers disclosed that the G' of 1.45 MPa, *n* = 6, increased after thrombin treatment to 2.05 MPa. This increase was attributed to conversion to fibrin of fg that had been incorporated on the fibril surface and increased lateral intrafibril and fiber contacts via knob A/hole a, αC/αC, and knob B/hole b contacts. Lastly, fiber assembly was initiated by lateral protofibril/protofibril contact, Figs. 2B and C , and was followed by protofibril/fiber or fine fibril/fiber wrap-around, Figs. 4E and F , likely enhancing fiber stiffness.

### Platelet Adhesion Experiments

**Fig. 5A and B. Fluorescent confocal microscopy images of mAb-treated FR fg adsorbed on PS Surface.**
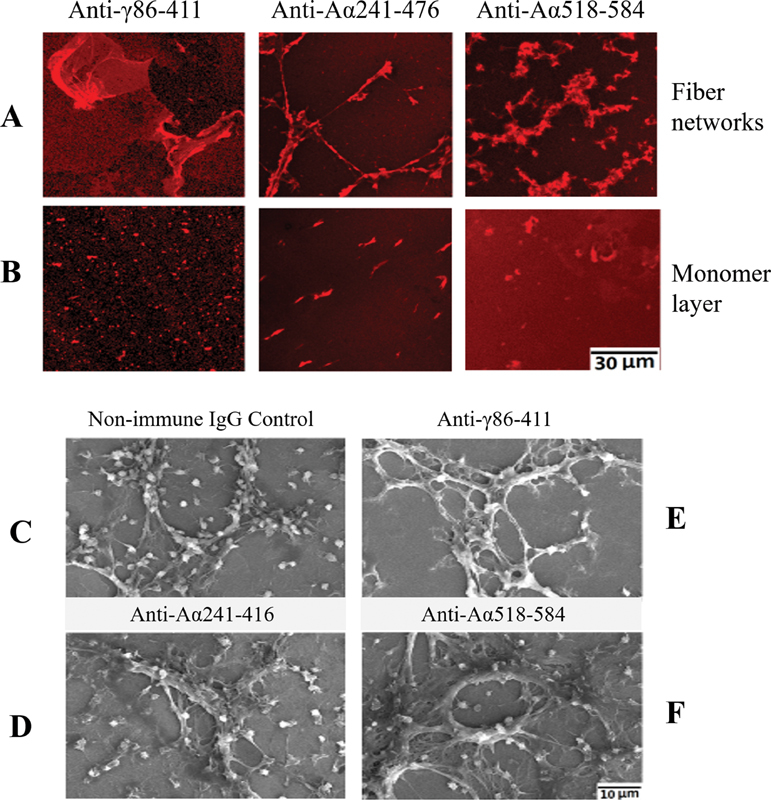
The bar and entry shown represents the magnification of all panels. ( **A** ) Each reflects a single experiment in which FR fg fiber networks, formed overnight were treated with each mAb shown (top) and subsequently with fluorescent anti-mouse IgG (see materials and methods). Note the dense signals by all three mAbs. ( **B** ) Nonfiber fg layer (see materials and methods) similarly treated with each mAb. This shows signals by the anti-γ86–411 mAb (left ), that are of variable size and sparse distribution and a faint punctate background. By contrast, the anti-Aα241–476 mAb (middle ) shows larger, mostly oblong, and less frequent signals suggesting the target epitope was exposed only on oligomeric forms. Anti-Aα518–584 mAb signals (right ) are similarly infrequent with of variable configuration. ( **C–F** ) **Scanning electron micrographs showing decreased platelets bound to fibers that had been pretreated with each mAb.** The Fibers generated on each PS surface wafer overnight were treated with anti-γ86–611, anti-Aα518–584, or anti-Aα241–476, as shown, prior to exposure to platelets whose liquid phase count was 2.83 × 10 ^5^ /μL. Platelet images appeared on or near fine or coarse fibers with least numbers on the E field. Using four different fields from each experiment, computed fiber-bound platelets/cm ^2^ were as follows: D 25%, E 11%, and F 31% relative to nonimmune IgG control fields, C. The bar and entry shown represents magnification in all panels. FD, fibrinogen depleted of soluble fibrin; Fg, fibrinogen; FR, fibrinogen enriched with soluble fibrin; MG, modified graphite; PS, polystyrene.

Reports of platelet adhesion to fg and fibrin under various conditions 40 41 42 43 led to our previous report describing platelet adhesion to adsorption-induced fg fibers in the plasma environment under both static and flow conditions 9_._ To explore exposed epitopes that bound platelets, we used three mAbs with broad specificity that included at least two known structures (e.g., Aα572–575, and the C-terminal dodecapeptide of the γ chain) that bind to the platelet integrin αIIbβ3 (IIb/IIIa). 44 To assess exposed epitopes, fiber networks and adsorbed monomers were exposed to each mAb and examined by confocal fluorescent microscopy. The results disclosed that target epitopes of all three mAbs, γ86–411, Aα241–476, and Aα518–584 were densely exposed on fibers ( Fig. 5A ). In sharp contrast, the nonfiber layer, Fig. 5B showed a pronounced sparsity of each epitope. We next examined platelet adhesion by scanning electron microscopy following glutaraldehyde fixation of immobilized platelets. The nonfiber layer displayed an occasional platelet as did PS controls lacking fg, and this was also indicated in spaces between fibers ( Fig. 5 ) and in several experiments not shown. In sharp contrast, fibers bound numerous platelets ( Fig. 5C ) with little shape change, if any. To assess binding further, fiber networks were pretreated with each mAb prior to exposure to platelets. As shown in Fig. 5D–F , each mAb markedly diminished platelet binding, relative to nonimmune mouse IgG control, Fig. 5C . That is, the number of platelets on treated fiber networks ranged from 11 to 31% of controls in one and 11 to 39% in a second experiment. This established that FR fg fibers displayed platelet adhesion in sharp contrast to adsorbed monomers.

### Investigations of Fiber Networks

**Fig. 6:**
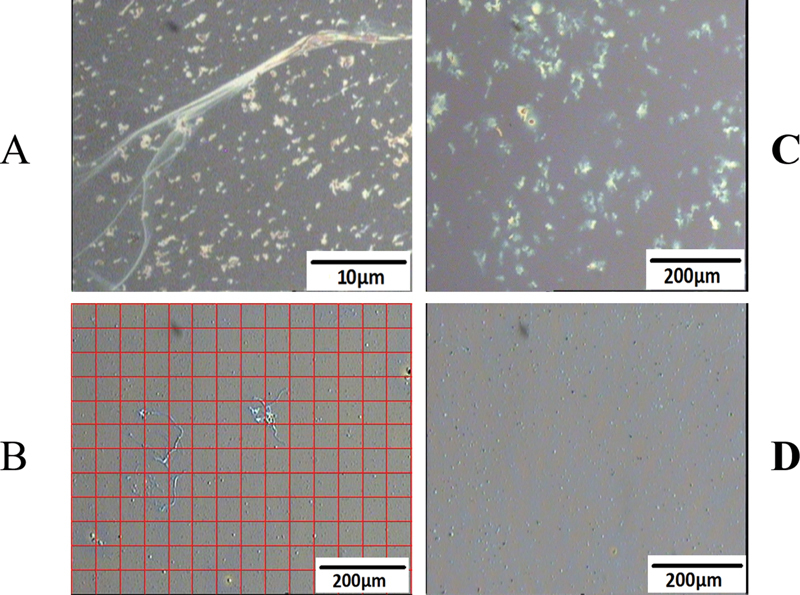
**PS surface fields of fiber patches generated following overnight adsorption.** ( **A** ) Fiber patches with a large overlying fiber generated by FR fg, 3 mg/mL. ( **B** ) An experiment showing rare fiber patches generated by FR fg on PS surface precoated with FD fg. The magnification bar relates to the field images and not to the overlying square grid used to quantify them. ( **C** ) An experiment showing fiber patches generated by FR fg on PS surface precoated with FR fg. ( **D** ) An experiment showing no generation of fiber patches by FR fg on PS surface that had been precoated with FR fg and then treated with fragment D _1_ and washed with buffer excess.

To quantify networks generated on PS surface, a grid of squares was superimposed 34 on a field of fiber patches identified by optical microscopy ( Figs. 6A , B , and C ). Expressed as %/cm ^2^ surface area containing fiber patches, FR fg patches amounted to 94.2 ± 2.7%, *n* = 4, compared of 5.3 ± 1.9% of FD fg counterparts. In general agreement, measurements of PS adsorbed fg/fibrin at 5-minute adsorption time (by a protein assay), 8 which disclosed 0.27 ± 0.03 μg/mm ^2^ surface area ( *n* = 4) for FR fg and 0.05 ± 0.012 μg/mm ^2^ for FD fg.

Optical microscopy was also used to assess generation of fiber patches under different adsorption conditions. In one set of experiments, FR fg applied to PS surfaced wafers (3 mg/mL, overnight) yielded numerous fiber patches with an overlying fiber ( Fig. 6A ). By contrast, FD fg yielded rare fiber patches, Fig. 6B . This field also shows a superimposed measurement grid. In a similar set of experiments, FR fg applied to its preformed monolayer also resulted in numerous fiber patches, Fig. 6C . However, when the preformed FR fg layer was exposed to (5 μM) fg fragment D _1_ (several hours) and washed subsequent application of FR fg ( Fig. 6D ) resulted in no patches, indicating that fragment D had blocked fiber generation. To explore this further, the preformed FR fg layer was exposed to 50 μM GPRP peptide (also for several hours) and washed. Subsequent application of FR fg also resulted in no fibers, not shown. In a further set of experiments a des-αC fg layer generated by adsorption to PS wafers was treated with thrombin, subsequently with PMSF (to inactivate thrombin, see materials and methods), and then exposed to 3 mg/mL FR fg. This too failed to generate fiber patches, *n* = 2, not shown. Together, these results established that neither knob/hole nor intermolecular αC/αC contacts per se could enable fiber generation induced by hydrophobic adsorption. By extension, they were both required and likely in a concerted sequence for this process.

## Discussion

The results established for the first time that fg fibers were entirely generated by its SF component. They also yielded evidence that the major SF component by far consisted of des-AA monomers that accounted for at least 80% of its protein (vide infra). Its remaining component, polymeric clusters, consisted of a protofibril core, indicated by crosslinking of their molecular constituents, in complex with fg. Variations of cluster diameter, moreover, likely reflected its protofibril length. The cluster presence in scanning electron micrographs of clots ( Fig. 1C–E ) may have reflected their incomplete participation owing to rapid polymerization (e.g., 1 U thrombin/mL). That is, αC/αC contacts may immobilize the cluster at a non-elongating site preventing its components from incorporation into the protofibril. Immobilization at an elongating site may dislodge cluster fg allowing the core to fully or partially unravel and incorporate in the protofibril.

Apart from the role by des-AA fibrin monomers in protofibril elongation (vide infra), three lines of evidence indicated a pivotal cluster role. First, protofibrils and fiber networks were routinely generated by (cluster rich) FR fg in sharp contrast to its (cluster poor) FD fg counterpart. Second, the thrombin experiments, ( Fig. 2H and I ) showing close links of protofibrils to clusters suggested cluster participation in protofibril elongation. Third, fiber networks were generated by a cluster concentrate, an FR subfraction, FRC ( Fig. 4H ) with minimal solitary monomer presence if any. These clusters generated numerous fine fibers that appeared to merge into coarse counterparts, a capacity that tempts the suggestion that the αC connector structure(s) that mediate lateral fibril assembly 8 was/were exposed on the clusters. Another role of the fg αC region was in fiber generation per se. We reported a clear association of this region with fine fibril elongation 14 which was entirely dependent on its αC domain part. 8 Also, both C domains of each des-AA monomer were critical for elongation. 8 Moreover, absence of protofibril images among des-αC fg clusters ( Fig. 2A ) tempted the suggestion that loss of cluster fg ( Fig. 4A and B ) possibly reflected adsorption via proximal sites of its αC region, 24 in addition to that via its D region. 8

Evidence of solitary des-AA fibrin monomers was provided by the large populations of cryoprecipitable monomers co-isolating with (cluster rich) FR fg and with cryoprecipitate fg ( Fig. 2D and F ). This interpretation is consistent with the decreased FpA of the parent fg. 2 37 Furthermore, the apparent mass of cluster populations in FR fg ( Fig. 4B ) seems insufficient to account for the substantial mass of generated fiber networks 8 ( Fig. 6A and C ). This discrepancy can be explained by the large des-AA monomer populations whose adsorption per se initiated protofibril assembly (vide infra).

Apart from our reported 8 and present evidence ( Fig. 2A ) that the αC region is essential for fiber generation, our results provided compelling evidence that knob/hole contacts were also essential. Specifically, the knob/hole interaction requirement was shown by inhibition experiments using fg fragment D _1_ ( Fig. 6D ), by the GPRP peptide, and by thrombin-treated des-αC fg (vice supra). In short, absence of either αC/αC or knob/hole contacts prevented protofibril generation intimating that both were required and were likely concerted participants.

While results from the foregoing time course experiments can explain the transient abundance of protofibrils and their elongation, they also argue that protofibrils elongated slowly in above surface directions and two factors limited the rate of their lateral self-assembly. One was that their elongation for some distance above surface enabled adequate length and proximity to one another resulting in lateral contact. Second, much if not most of their elongation was driven by des-AA monomers which lacked knob B/hole b contacts that enhance lateral protofibril assembly. What is more, the apparently slow unraveling of clusters by thrombin ( Fig. 2H and I ) raised the possibility that the reported distinct regions of network structure of thrombin induced clots of soluble fibrin 45 possibly reflect incompletely unraveled clusters and associated protofibrils that did not mature into normally organized fiber networks. Admittedly, our examples of such areas ( Figs. 2B , and 4H, I ) resulted from polymerization under nonprotease conditions, but they nevertheless offer a possible explanation which is in general agreement with the decreased G' of a thrombin induced FR clot (vide supra).

**Fig. 7:**
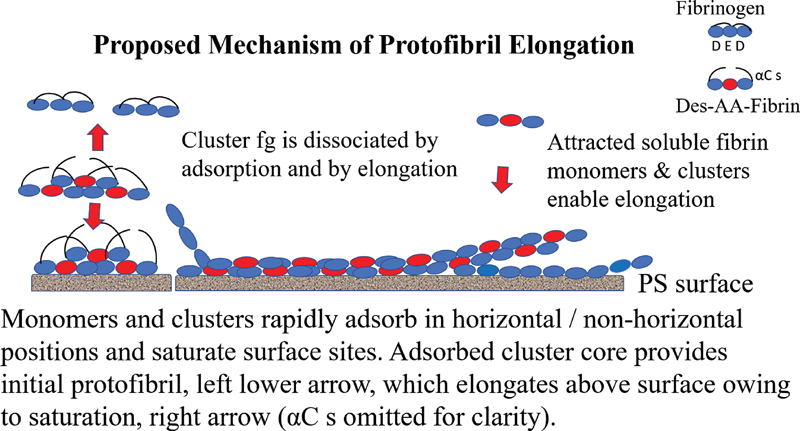
**Proposed mechanism of adsorption-induced protofibril generation.** Upper right area shows a monomer cartoon with its αCs tethered to its central (E) region. Below it is a des-AA fibrin monomer with its αC s partly untethered. The red nodule or fibrin E region indicates both its knobs A are exposed (des-AA fibrin. The left area shows two cluster cores without their fg, upper, and lower vertical arrows. The lower of the two is shown adsorbed to PS the surface. The middle area shows a protofibril (without its αC s for simplicity), which is elongating above the surface (right) owing to saturation of surface sites. Elongation is enabled by random capture of a new des-AA monomer, right arrow, and/or cluster, not shown, via “αC/αC” interactions that guide each into “knob A: hole a” docking. Fg is dislodged from the adsorbed cluster by (i) its high affinity for surface sites. Not illustrated are initial protofibrils assembled by adsorbed des-AA monomers that attracted counterparts from the overlying solution. Fg, fibrinogen; PS, polystyrene.

Evidence of adsorbed protofibrils which increased and elongated in time led to formulation of a possible mechanism illustrated in part in Fig. 7 . It suggests that cluster fg was dissociated during immobilization owing to its proximity and tight affinity to the hydrophobic surface 15 16 in part mediated via its αC region. 24 By extension, this was tighter than that of fg/protofibril (i.e. single knob/hole and possible αC/αC) contacts, while contacts of fibrin monomers self-assembled within the core were sufficiently tight to be unaffected. On a related observation mentioned previously, the marked protofibril abundance at 2-minute adsorption time ( Fig. 4A and B ) could not be accounted for by the limited cluster population ( Fig. 4B ). Consequently, adsorbed des-AA monomers served to additionally attract and immobilize clusters and dissociate their fg in addition to direct cluster adsorption to PS surface per se. This further enhanced elongation by randomly attracting counterparts and/or clusters from the overlying solution which were docked in place by knob/hole and αC/αC contacts. Together the foregoing can explain both the numerous initial protofibrils ( Fig. 4A and B ) and their subsequently increased length ( Fig. 4C and D ).

Our platelet adhesion results corroborate and in addition quantify those in our previous report. 9 The marked decrease of platelet adhesion to fibers by pretreatment with each mAb, anti-Aα518–584 and anti-γ86–411 reflected the exposure of fg/fibrin motifs that bind platelet αIIbβ3 (IIb/IIIa). 44 A similar decrease of adhesion by a third mAb, anti-fg Aα241–476, suggests possible allosteric effects or exposure of a cryptic site(s). Also, possible exposure of the Aα95–97 integrin recognition site may account for the minor fraction of platelets not blocked by any of the mAbs used. Lack of platelet adhesion to the non-fiber layer (vide supra) corroborates results by another group 41 also using monomers adsorbed to ostensibly hydrophobic surfaces. That is, both studies demonstrated that adsorbed fg monomers per se lacked platelet adhesion capacity.

The abundance of SF in plasma 1 2 argues for its generation mostly during phlebotomy and subsequent blood processing. However, its presence in circulation has been intimated by experimental evidence of rapid clearance des-AA monomers by macrophage uptake in a rabbit model. 46 Also, appreciable SF levels in blood collected via siliconized needles and tubing into hirudin or heparin anticoagulants (Galanakis, unpublished data) intimated that part of SF is of circulatory origin and is consistent with its reported abnormally elevated levels in various disorders. 3 Our results argue strongly, moreover, that clinical SF assays 3 as well cryoprecipitate quantitation 1 reflect mostly des-AA fibrin monomers, clusters comprising a significant but minor SF component. What is more, the functional properties of SF bespeak of its potential clinical relevance. One is its acceleration of thrombin-induced polymerization 37 (and vide supra). The other is that adsorption to the aforementioned surfaces does not impair the spontaneous coagulability of des-AA monomers or of clusters. The third is that the demonstrated adsorption induced fiber network generation in flowing plasma 9 serves to tightly anchor the resulting network to the underlying surfaces. What is more, the clear hemostasis-enhancing capacity of SF may promote pathologic thrombosis in the settings of its elevated levels following fg replacement therapy, 6 massive transfusions, 7 and SF adsorption to intravascular devices, 5 atheroma, and other vascular lesions. Lastly, the widely used clinical D-dimer assay, reflecting lysis of thrombin induced clots, makes revisiting SF measurements seem unattractive. However, our results suggest that most adsorbed SF (e.g. fiber networks generated on atheroma, metal, or hydrophobic intravascular device surfaces) is not crosslinked until thrombin is topically generated. Cluster measurements, therefore, may provide clinically useful information additional to that of D-dimer levels.

**Fig. 8:**
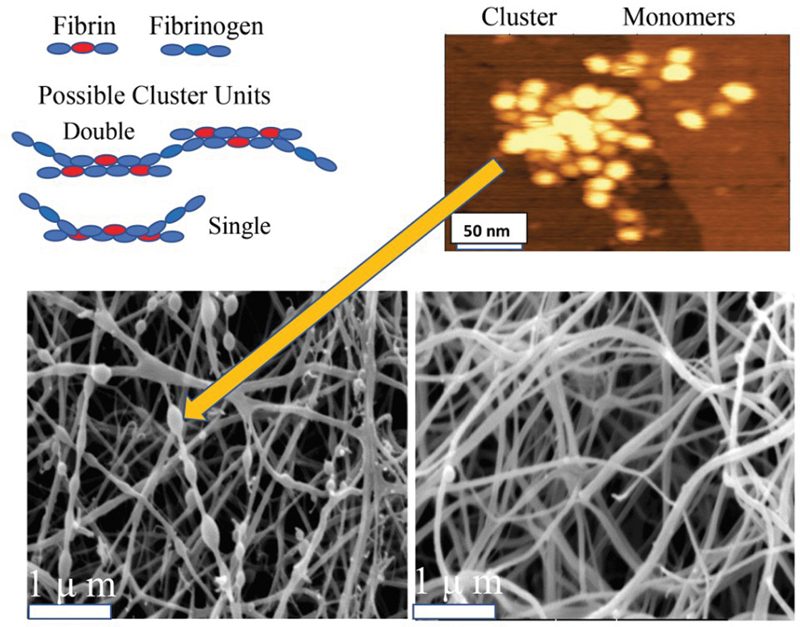
**Summary of cluster characteristics.** Upper Right: High resolution AFM scan showing monomer components of a cluster, repeated from Fig. 2E . Upper Left: schematic illustrations of two possible cluster configurations that can explain the variability of cluster size. The single unit cartoon implies variable protofibril elongation. The double unit reflects possible non-linear expansion. Both types of units are consistent with the invariably globular cluster shape, except for those incorporated in fibers that seem slightly stretched. Lower Left: scanning electron micrograph of a thrombin-induced clot of fg from a thrombophilic patient, repeated from Fig 1E , showing a substantial frequency of clusters among which those that appear embedded in fibers are ovoid or spindle shaped of (arrow). Lower Right: micrograph of a clot of fg from a normal donor showing a markedly lower cluster frequency (each migrograph magnification bar = 1 mm). Our data suggest that unraveling of clusters is favored by relatively low thrombin concentrations, Fig. 2H and I . Also, we postulate that cluster links to and incorporation in fibers are both αC mediated.
